# Bis­(μ-pyridine-2,3-dicarboxyl­ato)bis­[aqua­(3-carb­oxy­pyridine-2-carboxyl­ato)indium(III)] tetra­hydrate

**DOI:** 10.1107/S1600536811053566

**Published:** 2011-12-21

**Authors:** H. Eshtiagh-Hosseini, M. Mirzaei, A. Mousavinezhad, M. Necas, J. T. Mague

**Affiliations:** a Department of Chemistry, Ferdowsi University of Mashhad, 917791436 Mashhad, Iran; b Department of Chemistry, Faculty of Science, Masaryk University, Kamenice 5, Brno 625 00, Czech Republic; c Department of Chemistry, Tulane University, New Orleans, LA 70118, USA

## Abstract

In the binuclear centrosymmetric title compound, [In_2_(C_7_H_3_NO_4_)_2_(C_7_H_4_NO_4_)_2_(H_2_O)_2_]·4H_2_O, which contains both pyridine-2,3-dicarboxyl­ate and 3-carb­oxy­pyridine-2-carboxyl­ate ligands, the In^III^ atom is six-coordinated in a distorted octa­hedral geometry. One pyridine ligand is *N*,*O*-chelated while the other is *N*,*O*-chelated and at the same time bridging to the other *via* the second carboxyl group. In the crystal, an extensive O—H⋯O hydrogen-bonding network, involving the coordinated and lattice water mol­ecules and the carboxyl groups of the ligands, together with C—H⋯O and π–π inter­actions [centroid–centroid distance = 3.793 (1) Å], leads to the formation of a three-dimensional structure.

## Related literature

For metal complexes with polycarboxyl­ate ligands, see: Aghabozorg, Daneshvar *et al.* (2007 ▶); Aghabozorg, Khadivi *et al.* (2008 ▶); Aghabozorg, Ramezanipour *et al.* (2006 ▶); Eshtiagh-Hosseini *et al.* (2010 ▶); Mirzaei *et al.* (2011 ▶). For examples of self-assembly, see: Kondo *et al.* (1999 ▶); Beobide *et al.* (2006 ▶). For a discussion of hard–soft acid base concepts, see: Schlemper *et al.* (1967 ▶). For examples of π–π stacking, see: Janiak (2000 ▶). For three-dimensional network structures, see: Krygowski *et al.* (1998 ▶).
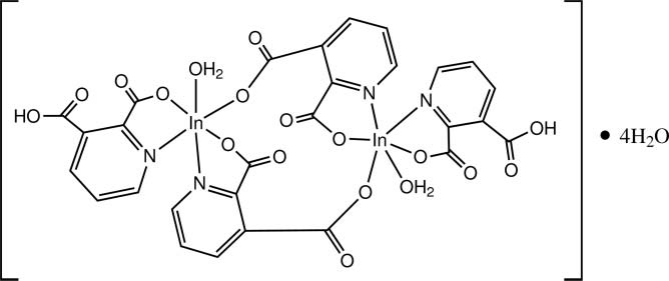


## Experimental

### 

#### Crystal data

[In_2_(C_7_H_3_NO_4_)_2_(C_7_H_4_NO_4_)_2_(H_2_O)_2_]·4H_2_O
*M* *_r_* = 1000.17
Triclinic, 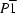

*a* = 8.0166 (3) Å
*b* = 10.0890 (4) Å
*c* = 11.9838 (5) Å
α = 110.069 (4)°
β = 96.236 (3)°
γ = 109.076 (3)°
*V* = 833.36 (6) Å^3^
*Z* = 1
Mo *K*α radiation
μ = 1.49 mm^−1^
*T* = 120 K
0.40 × 0.30 × 0.30 mm

#### Data collection

Oxford Diffraction Xcalibur diffractometer with a Sapphire2 detector
Absorption correction: multi-scan (*CrysAlis RED*; Oxford Diffraction, 2009 ▶) *T*_min_ = 0.588, *T*_max_ = 0.664
10774 measured reflections
3373 independent reflections
3168 reflections with *I* > 2σ(*I*)
*R*_int_ = 0.013

#### Refinement

*R*[*F* ^2^ > 2σ(*F* ^2^)] = 0.016
*wR*(*F* ^2^) = 0.039
*S* = 1.10
3373 reflections
254 parameters
H-atom parameters constrained
Δρ_max_ = 0.38 e Å^−3^
Δρ_min_ = −0.31 e Å^−3^

### 

Data collection: *CrysAlis CCD* (Oxford Diffraction, 2009 ▶); cell refinement: *CrysAlis CCD*; data reduction: *CrysAlis RED* (Oxford Diffraction, 2009 ▶); program(s) used to solve structure: *SHELXS97* (Sheldrick, 2008 ▶); program(s) used to refine structure: *SHELXL97* (Sheldrick, 2008 ▶); molecular graphics: *SHELXTL* (Sheldrick, 2008 ▶); software used to prepare material for publication: *SHELXTL*.

## Supplementary Material

Crystal structure: contains datablock(s) I, global. DOI: 10.1107/S1600536811053566/su2349sup1.cif

Structure factors: contains datablock(s) I. DOI: 10.1107/S1600536811053566/su2349Isup2.hkl

Additional supplementary materials:  crystallographic information; 3D view; checkCIF report

## Figures and Tables

**Table 1:** Hydrogen-bond geometry (Å, °)

*D*—H⋯*A*	*D*—H	H⋯*A*	*D*⋯*A*	*D*—H⋯*A*
O9—H91⋯O11^i^	0.84	1.75	2.5938 (19)	178
O9—H92⋯O3^i^	0.84	1.80	2.6402 (17)	175
O11—H112⋯O7^ii^	0.84	1.95	2.7595 (18)	162
O10—H101⋯O5^iii^	0.84	1.97	2.8065 (18)	175
O10—H102⋯O7^iv^	0.84	1.88	2.7237 (19)	178
O4—H4*O*⋯O10	0.84	1.67	2.5124 (17)	178
C4—H4⋯O1^v^	0.95	2.35	3.231 (2)	154
C5—H5⋯O6^vi^	0.95	2.36	3.293 (2)	168
C11—H11⋯O3^vii^	0.95	2.61	3.495 (2)	156
C12—H12⋯O2^vii^	0.95	2.33	2.993 (2)	126

Symmetry codes: (i) 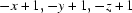; (ii) 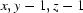; (iii) 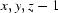; (iv) 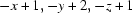; (v) 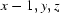; (vi) 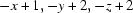; (vii) 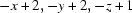.

**Fig. 1.**
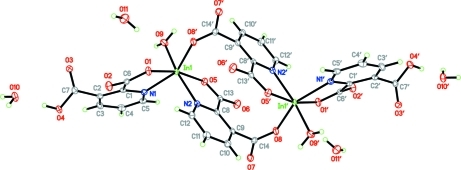
View of the local coordination environments of InIII atoms in the title molecule, with 50% probability thermal ellipsoids. Primed atoms are related to the non-primed atoms by the center of symmetry.

**Fig. 2.**
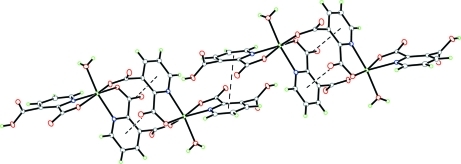
Perspective view of the π···π and C—O···π stacking interactions (dashed lines) in the title compound (In = large green circles, H = small green circles, O = red circles, N = blue circles).

**Fig. 3.**
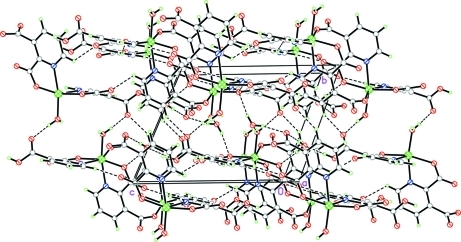
Perspective view of the crystal packing of the title compound, showing the intermolecular hydrogen bonds as dashed lines (In = large green circles, H = small green circles, O = red circles, N = blue circles; see Table 1 for details).

## supplementary crystallographic information

### Comment

For several years our research group, among others, has been interested in the synthesis of new coordination compounds from polycarboxylic acids and amines using a proton transfer methodology (Aghabozorg, Daneshvar, *et al.*, 2007; Aghabozorg, Khadivi, *et al.*, 2008; Aghabozorg, Ramezanipour, *et al.*, 2006; Eshtiagh-Hosseini *et al.*, 2010; Mirzaei *et al.*, 2011). Polycarboxylate ligands are versatile because of their diversity of coordination modes and also because of their ability to initiate self assembly processes by supramolecular interactions (Kondo *et al.*, 1999; Beobide *et al.*, 2006). In the majority of the complexes obtained by proton-transfer methods the metal complex is anionic with the cation derived from the amine used in the synthesis. Among these multicarboxylate ligands, pyridine-2,3-dicarboxylic acid (py-2,3dcH_2_) has rarely been used under the conditions generally employed in our studies. In the course of this work we prepared the title binuclear indium(II) compound, whose crystal structure we report on herein.

In addition to being a neutral complex, the title compound (Fig. 1) appears to be the first indium^III^ complex N,*O* chelated by one py-2,3-dcH^-^ ion and one py-2,3-dc^2-^ion, with the latter using the carboxyl group in the 3-position to bridge to a second metal. In the resulting centrosymmetric dimer, the coordination sphere of each metal is completed by a water molecule. The O_4_N_2_ coordination sphere adopts a distorted octahedral geometry with the largest departure being the 75.30 (5)° angles subtended by the chelating ligands (Fig. 1). The average In—O distance of 2.1253 (14) Å is slightly shorter than the average In—N of 2.2478 (17) Å. This can be explained by Pearson's hard and soft acid-base concept (Schlemper *et al.*, 1967).

The solid state structure can be described as chains of dimers associated *via* hydrogen bonding interactions between the coordinated water molecule, the monoprotonated carboxyl group and oxygen atoms in the pyridine dicarboxylate ligand as well as C—H···O interactons between ring hydrogen atoms and carboxylate oxygen atoms (Table 1). Additionally there is a slipped π-π stacking interaction (Fig. 2) between the (N1,C1-C5) ring and its counterpart in the dimer at -*x+1*, -*y+2*, -*z+1* [perpendicular separation = 3.107 (1) Å, centroid-to-centroid distance = 3.793 (1) Å, slippage = 1.37 Å, angle between planes = 11.28 (8)° (Janiak, 2000)]. The chains are associated *via* hydrogen bonding interactions between the lattice water molecules, the coordinated water molecule and oxygen atoms of the carboxylate ligands (Table 1, Fig. 3) to complete the three-dimensional network structure (Krygowski *et al.*, 1998).

A final interaction of significance is a complementary π-π stacking interaction (Fig. 2) between the C13═O6 moiety in one half of the dimer and the (N2,C8-C12) ring in the other half (centroid-to-centroid distance = 3.347 (2) Å, angle of the line joining the centroids to the plane of the ring = 74.7 (1)°).

### Experimental

A solution of In_2_(SO_4_)_3_.xH_2_O (34 mg, 0.06 mmol) in water (5 ml) was added dropwise to an aqueous solution of pyridine-2,3-dicarboxylic acid (10 mg, 0.06 mmol) and 2-amino-6-methyl pyridine (13 mg, 0.12 mmol) in a 1:1:2 molar ratio at reflux. The solution was cooled to room temperature and upon slow evaporation, X-ray quality crystals were formed which were collected and washed with distilled water.

### Refinement

The OH and water H-atoms were located in difference Fourier maps and were refined as riding atoms with U_iso_(H) = 1.2U_eq_(O). The C-bound H-atoms were placed in calculated positions and treated as riding atoms: C—H = 0.95 Å with U_iso_(H) = 1.2U_eq_(C).

### Crystal data

**Table:**

[In_2_(C_7_H_3_NO_4_)_2_(C_7_H_4_NO_4_)_2_(H_2_O)_2_]·4H_2_O	*Z* = 1
*M**_r_* = 1000.17	*F*(000) = 496
Triclinic, *P*1	*D*_x_ = 1.993 Mg m^−^^3^
Hall symbol: -P 1	Mo *K*α radiation, λ = 0.71073 Å
*a* = 8.0166 (3) Å	Cell parameters from 8569 reflections
*b* = 10.0890 (4) Å	θ = 2.8–27.2°
*c* = 11.9838 (5) Å	µ = 1.49 mm^−^^1^
α = 110.069 (4)°	*T* = 120 K
β = 96.236 (3)°	Block, colourless
γ = 109.076 (3)°	0.40 × 0.30 × 0.30 mm
*V* = 833.36 (6) Å^3^	

### Data collection

**Table:**

Oxford Diffraction Xcalibur diffractometer with a Sapphire2 detector	3373 independent reflections
Radiation source: Enhance (Mo) X-ray Source	3168 reflections with *I* > 2σ(*I*)
graphite	*R*_int_ = 0.013
Detector resolution: 8.4353 pixels mm^-1^	θ_max_ = 27.2°, θ_min_ = 3.3°
ω scan	*h* = −8→10
Absorption correction: multi-scan (*CrysAlis RED*; Oxford Diffraction, 2009)	*k* = −12→12
*T*_min_ = 0.588, *T*_max_ = 0.664	*l* = −15→15
10774 measured reflections	

### Refinement

**Table:**

Refinement on *F*^2^	Secondary atom site location: difference Fourier map
Least-squares matrix: full	Hydrogen site location: inferred from neighbouring sites
*R*[*F*^2^ > 2σ(*F*^2^)] = 0.016	H-atom parameters constrained
*wR*(*F*^2^) = 0.039	*w* = 1/[σ^2^(*F*_o_^2^) + (0.0197*P*)^2^ + 0.3217*P*] where *P* = (*F*_o_^2^ + 2*F*_c_^2^)/3
*S* = 1.10	(Δ/σ)_max_ = 0.002
3373 reflections	Δρ_max_ = 0.38 e Å^−^^3^
254 parameters	Δρ_min_ = −0.31 e Å^−^^3^
0 restraints	Extinction correction: *SHELXL97* (Sheldrick, 2008), Fc^*^=kFc[1+0.001xFc^2^λ^3^/sin(2θ)]^-1/4^
Primary atom site location: structure-invariant direct methods	Extinction coefficient: 0.0083 (5)

### Special details

**Table:**

Geometry. All e.s.d.'s (except the e.s.d. in the dihedral angle between two l.s. planes) are estimated using the full covariance matrix. The cell e.s.d.'s are taken into account individually in the estimation of e.s.d.'s in distances, angles and torsion angles; correlations between e.s.d.'s in cell parameters are only used when they are defined by crystal symmetry. An approximate (isotropic) treatment of cell e.s.d.'s is used for estimating e.s.d.'s involving l.s. planes.
Refinement. Refinement of *F*^2^ against ALL reflections. The weighted *R*-factor *wR* and goodness of fit *S* are based on *F*^2^, conventional *R*-factors *R* are based on *F*, with *F* set to zero for negative *F*^2^. The threshold expression of *F*^2^ > σ(*F*^2^) is used only for calculating *R*-factors(gt) *etc*. and is not relevant to the choice of reflections for refinement. *R*-factors based on *F*^2^ are statistically about twice as large as those based on *F*, and *R*- factors based on ALL data will be even larger.

### Fractional atomic coordinates and isotropic or equivalent isotropic displacement parameters (Å^2^)

**Table:**

	*x*	*y*	*z*	*U*_iso_*/*U*_eq_	
In1	0.716069 (17)	0.792788 (13)	0.730349 (10)	0.00941 (5)	
O1	0.81082 (17)	0.78122 (15)	0.57022 (11)	0.0132 (3)	
O2	0.77166 (18)	0.81433 (15)	0.39645 (11)	0.0165 (3)	
O3	0.43118 (19)	0.60530 (14)	0.22385 (11)	0.0154 (3)	
O4	0.38691 (19)	0.80920 (15)	0.21595 (11)	0.0179 (3)	
H4O	0.3870	0.7721	0.1416	0.021*	
O5	0.64775 (17)	0.86624 (14)	0.90207 (10)	0.0127 (3)	
O6	0.73152 (19)	1.05169 (15)	1.08983 (11)	0.0171 (3)	
O7	0.92356 (19)	1.40964 (15)	1.17662 (11)	0.0165 (3)	
O8	1.10615 (18)	1.31541 (14)	1.24362 (11)	0.0144 (3)	
O9	0.50749 (18)	0.56958 (14)	0.66940 (11)	0.0168 (3)	
H91	0.3972	0.5539	0.6473	0.020*	
H92	0.5325	0.5180	0.7057	0.020*	
N1	0.5111 (2)	0.83155 (16)	0.61329 (13)	0.0105 (3)	
N2	0.9159 (2)	1.03454 (17)	0.83361 (13)	0.0104 (3)	
C1	0.5347 (2)	0.80161 (19)	0.49914 (15)	0.0101 (4)	
C2	0.4018 (3)	0.78340 (19)	0.40403 (16)	0.0120 (4)	
C3	0.2455 (3)	0.8044 (2)	0.43128 (17)	0.0159 (4)	
H3	0.1527	0.7938	0.3685	0.019*	
C4	0.2254 (3)	0.8406 (2)	0.54992 (17)	0.0165 (4)	
H4	0.1209	0.8583	0.5699	0.020*	
C5	0.3595 (3)	0.8508 (2)	0.63908 (16)	0.0135 (4)	
H5	0.3442	0.8717	0.7200	0.016*	
C6	0.7195 (2)	0.79807 (19)	0.48430 (15)	0.0107 (4)	
C7	0.4120 (3)	0.7255 (2)	0.27281 (16)	0.0124 (4)	
C8	0.9017 (3)	1.0999 (2)	0.94915 (15)	0.0100 (3)	
C9	1.0201 (3)	1.2475 (2)	1.02728 (15)	0.0113 (4)	
C10	1.1540 (3)	1.3289 (2)	0.98329 (16)	0.0153 (4)	
H10	1.2361	1.4305	1.0348	0.018*	
C11	1.1667 (3)	1.2612 (2)	0.86462 (16)	0.0158 (4)	
H11	1.2570	1.3154	0.8335	0.019*	
C12	1.0452 (3)	1.1130 (2)	0.79220 (16)	0.0131 (4)	
H12	1.0537	1.0655	0.7107	0.016*	
C13	0.7493 (3)	1.0014 (2)	0.98602 (15)	0.0113 (4)	
C14	1.0119 (3)	1.3270 (2)	1.15813 (15)	0.0121 (4)	
O10	0.39345 (18)	0.69562 (15)	−0.00496 (11)	0.0167 (3)	
H101	0.4704	0.7512	−0.0293	0.020*	
H102	0.2944	0.6616	−0.0572	0.020*	
O11	0.83456 (19)	0.48403 (16)	0.39983 (12)	0.0217 (3)	
H111	0.8943	0.5668	0.4609	0.026*	
H112	0.8849	0.4755	0.3413	0.026*	

### Atomic displacement parameters (Å^2^)

**Table:**

	*U*^11^	*U*^22^	*U*^33^	*U*^12^	*U*^13^	*U*^23^
In1	0.00961 (8)	0.01133 (8)	0.00717 (7)	0.00362 (6)	0.00213 (5)	0.00400 (5)
O1	0.0104 (7)	0.0217 (7)	0.0097 (6)	0.0077 (6)	0.0033 (5)	0.0072 (5)
O2	0.0164 (8)	0.0241 (7)	0.0127 (6)	0.0077 (6)	0.0072 (6)	0.0105 (6)
O3	0.0209 (8)	0.0118 (6)	0.0123 (6)	0.0055 (6)	0.0014 (6)	0.0050 (5)
O4	0.0275 (9)	0.0184 (7)	0.0112 (6)	0.0112 (6)	0.0027 (6)	0.0084 (5)
O5	0.0112 (7)	0.0143 (6)	0.0108 (6)	0.0026 (6)	0.0040 (5)	0.0049 (5)
O6	0.0181 (8)	0.0196 (7)	0.0113 (6)	0.0046 (6)	0.0074 (6)	0.0049 (5)
O7	0.0191 (8)	0.0186 (7)	0.0129 (6)	0.0119 (6)	0.0014 (6)	0.0039 (5)
O8	0.0164 (8)	0.0191 (7)	0.0098 (6)	0.0102 (6)	0.0018 (5)	0.0055 (5)
O9	0.0132 (7)	0.0150 (7)	0.0218 (7)	0.0033 (6)	−0.0003 (6)	0.0106 (6)
N1	0.0104 (8)	0.0097 (7)	0.0111 (7)	0.0032 (6)	0.0033 (6)	0.0046 (6)
N2	0.0103 (8)	0.0124 (7)	0.0098 (7)	0.0047 (7)	0.0026 (6)	0.0054 (6)
C1	0.0104 (10)	0.0068 (8)	0.0122 (8)	0.0017 (7)	0.0028 (7)	0.0044 (7)
C2	0.0130 (10)	0.0083 (8)	0.0135 (8)	0.0021 (8)	0.0013 (7)	0.0056 (7)
C3	0.0128 (10)	0.0173 (9)	0.0183 (9)	0.0062 (8)	0.0001 (8)	0.0087 (8)
C4	0.0124 (10)	0.0161 (9)	0.0238 (10)	0.0074 (8)	0.0067 (8)	0.0089 (8)
C5	0.0145 (10)	0.0109 (9)	0.0158 (9)	0.0048 (8)	0.0068 (8)	0.0057 (7)
C6	0.0100 (10)	0.0088 (8)	0.0100 (8)	0.0015 (7)	0.0013 (7)	0.0023 (7)
C7	0.0072 (9)	0.0135 (9)	0.0136 (8)	0.0008 (8)	−0.0013 (7)	0.0063 (7)
C8	0.0104 (10)	0.0131 (9)	0.0094 (8)	0.0063 (8)	0.0027 (7)	0.0062 (7)
C9	0.0115 (10)	0.0142 (9)	0.0100 (8)	0.0068 (8)	0.0012 (7)	0.0058 (7)
C10	0.0149 (11)	0.0139 (9)	0.0139 (8)	0.0027 (8)	0.0007 (8)	0.0054 (7)
C11	0.0147 (11)	0.0171 (9)	0.0153 (9)	0.0027 (8)	0.0056 (8)	0.0090 (8)
C12	0.0142 (10)	0.0158 (9)	0.0107 (8)	0.0057 (8)	0.0050 (7)	0.0065 (7)
C13	0.0104 (10)	0.0148 (9)	0.0113 (8)	0.0070 (8)	0.0017 (7)	0.0067 (7)
C14	0.0103 (10)	0.0110 (9)	0.0115 (8)	0.0009 (8)	0.0015 (7)	0.0038 (7)
O10	0.0133 (7)	0.0224 (7)	0.0134 (6)	0.0027 (6)	0.0014 (5)	0.0106 (6)
O11	0.0204 (8)	0.0215 (7)	0.0203 (7)	0.0069 (6)	0.0059 (6)	0.0061 (6)

### Geometric parameters (Å, °)

**Table:**

In1—O8^i^	2.1153 (12)	C1—C2	1.390 (2)
In1—O1	2.1199 (12)	C1—C6	1.522 (2)
In1—O9	2.1319 (13)	C2—C3	1.392 (3)
In1—O5	2.1324 (11)	C2—C7	1.500 (2)
In1—N2	2.2339 (15)	C3—C4	1.383 (3)
In1—N1	2.2618 (14)	C3—H3	0.9500
O1—C6	1.289 (2)	C4—C5	1.383 (3)
O2—C6	1.216 (2)	C4—H4	0.9500
O3—C7	1.221 (2)	C5—H5	0.9500
O4—C7	1.302 (2)	C8—C9	1.386 (3)
O4—H4O	0.8401	C8—C13	1.517 (3)
O5—C13	1.300 (2)	C9—C10	1.396 (3)
O6—C13	1.216 (2)	C9—C14	1.517 (2)
O7—C14	1.240 (2)	C10—C11	1.382 (2)
O8—C14	1.268 (2)	C10—H10	0.9500
O8—In1^i^	2.1152 (12)	C11—C12	1.383 (3)
O9—H91	0.8400	C11—H11	0.9500
O9—H92	0.8400	C12—H12	0.9500
N1—C5	1.343 (2)	O10—H101	0.8400
N1—C1	1.345 (2)	O10—H102	0.8400
N2—C12	1.339 (2)	O11—H111	0.8400
N2—C8	1.350 (2)	O11—H112	0.8400
			
O8^i^—In1—O1	83.58 (5)	C2—C3—H3	120.0
O8^i^—In1—O9	84.44 (5)	C5—C4—C3	119.06 (17)
O1—In1—O9	101.63 (5)	C5—C4—H4	120.5
O8^i^—In1—O5	104.07 (5)	C3—C4—H4	120.5
O1—In1—O5	165.16 (5)	N1—C5—C4	121.17 (16)
O9—In1—O5	91.87 (5)	N1—C5—H5	119.4
O8^i^—In1—N2	97.32 (5)	C4—C5—H5	119.4
O1—In1—N2	91.20 (5)	O2—C6—O1	125.21 (17)
O9—In1—N2	167.17 (5)	O2—C6—C1	119.13 (15)
O5—In1—N2	75.36 (5)	O1—C6—C1	115.64 (14)
O8^i^—In1—N1	153.16 (5)	O3—C7—O4	124.16 (16)
O1—In1—N1	75.34 (5)	O3—C7—C2	121.80 (15)
O9—In1—N1	83.75 (5)	O4—C7—C2	113.89 (15)
O5—In1—N1	100.33 (5)	N2—C8—C9	121.27 (16)
N2—In1—N1	99.52 (5)	N2—C8—C13	115.55 (15)
C6—O1—In1	119.19 (11)	C9—C8—C13	123.18 (15)
C7—O4—H4O	112.4	C8—C9—C10	118.45 (16)
C13—O5—In1	118.93 (11)	C8—C9—C14	123.82 (16)
C14—O8—In1^i^	140.24 (11)	C10—C9—C14	117.73 (16)
In1—O9—H91	121.2	C11—C10—C9	119.83 (18)
In1—O9—H92	112.4	C11—C10—H10	120.1
H91—O9—H92	117.5	C9—C10—H10	120.1
C5—N1—C1	120.12 (15)	C10—C11—C12	118.58 (18)
C5—N1—In1	126.80 (11)	C10—C11—H11	120.7
C1—N1—In1	111.76 (11)	C12—C11—H11	120.7
C12—N2—C8	119.97 (16)	N2—C12—C11	121.89 (16)
C12—N2—In1	126.11 (11)	N2—C12—H12	119.1
C8—N2—In1	113.89 (12)	C11—C12—H12	119.1
N1—C1—C2	121.65 (16)	O6—C13—O5	124.90 (17)
N1—C1—C6	115.19 (15)	O6—C13—C8	119.05 (16)
C2—C1—C6	123.09 (15)	O5—C13—C8	116.05 (14)
C1—C2—C3	118.00 (16)	O7—C14—O8	123.27 (15)
C1—C2—C7	122.31 (16)	O7—C14—C9	117.88 (15)
C3—C2—C7	119.33 (16)	O8—C14—C9	118.63 (15)
C4—C3—C2	119.91 (17)	H101—O10—H102	105.0
C4—C3—H3	120.0	H111—O11—H112	111.8
			
O8^i^—In1—O1—C6	162.67 (13)	C2—C3—C4—C5	−2.0 (3)
O9—In1—O1—C6	79.72 (13)	C1—N1—C5—C4	−0.1 (3)
O5—In1—O1—C6	−75.3 (2)	In1—N1—C5—C4	−165.89 (13)
N2—In1—O1—C6	−100.10 (13)	C3—C4—C5—N1	2.5 (3)
N1—In1—O1—C6	−0.57 (12)	In1—O1—C6—O2	169.57 (14)
O8^i^—In1—O5—C13	89.72 (12)	In1—O1—C6—C1	−8.69 (19)
O1—In1—O5—C13	−30.0 (2)	N1—C1—C6—O2	−159.83 (16)
O9—In1—O5—C13	174.45 (12)	C2—C1—C6—O2	16.9 (3)
N2—In1—O5—C13	−4.31 (11)	N1—C1—C6—O1	18.5 (2)
N1—In1—O5—C13	−101.56 (12)	C2—C1—C6—O1	−164.68 (16)
O8^i^—In1—N1—C5	138.04 (14)	C1—C2—C7—O3	53.6 (3)
O1—In1—N1—C5	177.42 (15)	C3—C2—C7—O3	−119.3 (2)
O9—In1—N1—C5	73.64 (15)	C1—C2—C7—O4	−130.59 (18)
O5—In1—N1—C5	−17.12 (15)	C3—C2—C7—O4	56.5 (2)
N2—In1—N1—C5	−93.83 (15)	C12—N2—C8—C9	−0.6 (2)
O8^i^—In1—N1—C1	−28.73 (19)	In1—N2—C8—C9	177.46 (12)
O1—In1—N1—C1	10.65 (11)	C12—N2—C8—C13	179.38 (15)
O9—In1—N1—C1	−93.12 (12)	In1—N2—C8—C13	−2.56 (18)
O5—In1—N1—C1	176.11 (12)	N2—C8—C9—C10	0.9 (2)
N2—In1—N1—C1	99.40 (12)	C13—C8—C9—C10	−179.06 (16)
O8^i^—In1—N2—C12	78.74 (14)	N2—C8—C9—C14	−179.73 (16)
O1—In1—N2—C12	−4.94 (14)	C13—C8—C9—C14	0.3 (3)
O9—In1—N2—C12	175.85 (19)	C8—C9—C10—C11	−0.5 (3)
O5—In1—N2—C12	−178.56 (15)	C14—C9—C10—C11	−179.94 (16)
N1—In1—N2—C12	−80.27 (14)	C9—C10—C11—C12	−0.1 (3)
O8^i^—In1—N2—C8	−99.18 (12)	C8—N2—C12—C11	−0.1 (3)
O1—In1—N2—C8	177.14 (12)	In1—N2—C12—C11	−177.90 (13)
O9—In1—N2—C8	−2.1 (3)	C10—C11—C12—N2	0.4 (3)
O5—In1—N2—C8	3.52 (11)	In1—O5—C13—O6	−174.77 (13)
N1—In1—N2—C8	101.80 (12)	In1—O5—C13—C8	4.34 (18)
C5—N1—C1—C2	−2.7 (3)	N2—C8—C13—O6	178.18 (15)
In1—N1—C1—C2	165.07 (13)	C9—C8—C13—O6	−1.8 (3)
C5—N1—C1—C6	174.13 (15)	N2—C8—C13—O5	−1.0 (2)
In1—N1—C1—C6	−18.10 (17)	C9—C8—C13—O5	179.00 (15)
N1—C1—C2—C3	3.0 (3)	In1^i^—O8—C14—O7	−176.98 (13)
C6—C1—C2—C3	−173.52 (16)	In1^i^—O8—C14—C9	−2.6 (3)
N1—C1—C2—C7	−169.95 (16)	C8—C9—C14—O7	−93.8 (2)
C6—C1—C2—C7	13.5 (3)	C10—C9—C14—O7	85.6 (2)
C1—C2—C3—C4	−0.7 (3)	C8—C9—C14—O8	91.5 (2)
C7—C2—C3—C4	172.56 (17)	C10—C9—C14—O8	−89.1 (2)

Symmetry codes: (i) −*x*+2, −*y*+2, −*z*+2.

### Hydrogen-bond geometry (Å, °)

**Table:**

*D*—H···*A*	*D*—H	H···*A*	*D*···*A*	*D*—H···*A*
O9—H91···O11^ii^	0.84	1.75	2.5938 (19)	178
O9—H92···O3^ii^	0.84	1.80	2.6402 (17)	175
O11—H112···O7^iii^	0.84	1.95	2.7595 (18)	162
O10—H101···O5^iv^	0.84	1.97	2.8065 (18)	175
O10—H102···O7^v^	0.84	1.88	2.7237 (19)	178
O4—H4O···O10	0.84	1.67	2.5124 (17)	178
C4—H4···O1^vi^	0.95	2.35	3.231 (2)	154
C5—H5···O6^vii^	0.95	2.36	3.293 (2)	168
C11—H11···O3^viii^	0.95	2.61	3.495 (2)	156
C12—H12···O2^viii^	0.95	2.33	2.993 (2)	126

Symmetry codes: (ii) −*x*+1, −*y*+1, −*z*+1; (iii) *x*, *y*−1, *z*−1; (iv) *x*, *y*, *z*−1; (v) −*x*+1, −*y*+2, −*z*+1; (vi) *x*−1, *y*, *z*; (vii) −*x*+1, −*y*+2, −*z*+2; (viii) −*x*+2, −*y*+2, −*z*+1.
